# Cardiovascular Changes Related to Metabolic Syndrome: Evidence in Obese Zucker Rats

**DOI:** 10.3390/ijms21062035

**Published:** 2020-03-16

**Authors:** Ilenia Martinelli, Daniele Tomassoni, Michele Moruzzi, Proshanta Roy, Carlo Cifani, Francesco Amenta, Seyed Khosrow Tayebati

**Affiliations:** 1 School of Pharmacy; University of Camerino, 62032 Camerino, Italy; ilenia.martinelli@unicam.it (I.M.); carlo.cifani@unicam.it (C.C.); francesco.amenta@unicam.it (F.A.); 2 School of Biosciences and Veterinary Medicine, University of Camerino, 62032 Camerino, Italy; daniele.tomassoni@unicam.it (D.T.); proshanta.roy@studenti.unicam.it (P.R.); 3 Department of Medicine, University of Leipzig, 04103 Leipzig, Germany; Michele.Moruzzi@medizin.uni-leipzig.de

**Keywords:** heart, inflammation, obesity, oxidative stress, Zucker rats

## Abstract

Metabolic syndrome (MetS) is a predictor of cardiovascular diseases, commonly associated with oxidative stress and inflammation. However, the pathogenic mechanisms are not yet fully elucidated. The aim of the study is to evaluate the oxidative status and inflammation in the heart of obese Zucker rats (OZRs) and lean Zucker rats (LZRs) at different ages. Morphological and morphometric analyses were performed in the heart. To study the oxidative status, the malondialdehyde (MDA), 4-hydroxynonenal (4-HNE), protein oxidation, and antioxidant enzymes were measured in plasma and heart. To elucidate the inflammatory markers involved, immunohistochemistry and Western blot were performed for cellular adhesion molecules and proinflammatory cytokines. OZRs were characterized by hypertension, hyperlipidemia, hyperglycemia, and insulin resistance. The obesity increased MDA and decreased the activities of superoxide dismutase (SOD) in plasma as well as in the heart, associated with cardiomyocytes hypertrophy. OxyBlot in plasma and in heart showed an increase of oxidativestate proteins in OZRs. Vascular cell adhesion molecule-1, interleukin-6, and tumor necrosis factor-α expressions in OZRs were higher than those of LZRs. However, these processes did not induce apoptosis or necrosis of cardiomyocytes. Thus, MetS induces the lipid peroxidation and decreased antioxidant defense that leads to heart tissue changes and coronary inflammation.

## 1. Introduction

Obesity is a chronic pathological condition with an accumulation of adipose tissue [1]. It has been documented as a primary factor in the pathogenesis for several diseases [2,3]. Visceral obesity is the central-causal component of metabolic syndrome (MetS) [4].

MetS groups a series of metabolic and cardiovascular risk factors such as impaired glucose tolerance, dyslipidemia, and hypertension that increase the hazard of type 2 diabetes mellitus and cardiovascular diseases (CVD) [5,6]. MetS is a challenge for modern medicine: it grows exponentially in adults, as well as in children and adolescents, and proportionally with changes in lifestyle [4].

The obesity and MetS effects on coronary microvascular and cardiovascular dysfunction are already reported and updated [7,8], even though the pathophysiology underlying this evidence is extremely complex and multifactorial [9]. Among the hypothesized mechanisms, insulin resistance (IR), neurohormonal activation, and low-grade systemic inflammation represent the main events involved in the evolution from obesity and/or MetS to CVD [10,11]. In obesity, epicardial fat accumulates and it changes its biological characteristics. It adopts many of the features of white adipose tissue, whose inclination to lipolysis leads to the release of fatty acids and reactive inflammation. Epicardial fat is a source of adiponectin and adrenomedullin, adipokines with anti-inflammatory properties, and several proinflammatory cytokines as tumor necrosis factor-alpha (TNF-α), interleukin-1 beta (IL-1β), interleukin-6 (IL-6), monocyte chemoattractiveprotein-1, nerve growth factor, resistin, plasminogen activator inhibitor-1, and free fatty acids. Epicardial adipose tissue could locally moderate the heart and vasculature, through paracrine secretion of pro- and anti-inflammatory cytokines, thereby playing a possible role in the adiposity-related inflammation and atherosclerosis [12,13]. Indeed, obese animals and people exhibited high levels of TNF-α, IL-1β, and IL-6, all produced by macrophages derived from adipose tissue [14], in which endothelial cells increased adhesion proteins, such as intercellular cell adhesion molecule-1 (ICAM-1), vascular cell adhesion molecule-1 (VCAM-1), and E-selectin [15]. Adhesion molecules play a central role in adherence of cells to endothelial surfaces, in the integrity of the vascular wall and can be modulated by dietary pattern and body composition [16].

Obesity is characterized not only by exacerbated inflammatory outcomes, but also by permanently increased oxidative stress [17,18]. This imbalance between antioxidant and pro-oxidant factors is strongly related to pro-inflammatory processes [19,20], leading to the development of obesity-related complications, atherosclerosis, and CVD [9,21,22]. Mitochondria are the primary source of reactive oxygen species (ROS). Their production and oxidative damage may contribute to the onset and progression of CVD, obesity, diabetes, and atherosclerosis [23].

The synthesis of ROS promoted an inflammatory status and dysregulated the expression of inflammation-associated adipocytokines in MetS, contributing to obesity-related cardiovascular risk through endothelial dysfunction and platelet activation [24,25,26].

As described above, the relation between oxidative stress and inflammation in MetS is crucial. It is still unclear how these simultaneous risk factors produce the variety of obesity-associated adverse CVD.

Although there is different data regarding the metabolic alterations in obese rats and the development of cardiac changes [27,28,29], no clear evidence concerning obesity-related oxidative stress and inflammation in the heart is present. Thus, investigating the interplay between these risk factors with obesity/MetS onset as well as the interactions with CVD is fundamental.

Therefore, this study was designed to evaluate changes in blood parameters, blood pressure, and the possible correlation between the development of the cardiac alterations and the onset of the oxidative stress and inflammatory processes in obese (fa/fa) Zucker rats (OZRs) as a model of MetS for the concomitant manifestation of obesity, hyperglycemia, hyperinsulinemia, hyperlipidemia, and moderate hypertension [30,31], compared to the littermate lean Zucker rats (LZRs).

## 2. Results

### 2.1. Physiological and Blood Parameters

As shown in Table 1, the body weight of obese rats was significantly higher than lean littermates, starting from 12 weeks of age and the amount of food eaten by the OZRs was higher. The systolic blood pressure measured the day of sacrifice, was significantly higher in 16 and 20 weeks old OZRs in comparison to LZRs (Table 1). The heart weight was remarkably increased in obese compared to lean groups at different ages (Table 1). In the OZRs at different ages, the open-field test revealed a decrease of cumulative distance traveled, with an increment of total immobility time (unpublished data).

Serum analyses of samples collected at 12, 16, 20 weeks of age showed that the values of glucose and insulin were higher in OZRs than in LZRs in all weeks. In addition to higher triglycerides levels, total-, LDL-, and HDL-cholesterol rose in proportion to the age in the obese animals (Table 1).

### 2.2. Oxidative Stress

**Figure 1:**
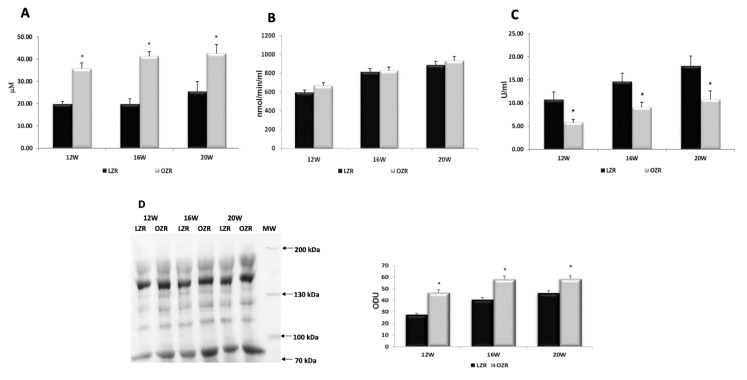
Oxidative stress in plasma. (**A**) Concentration of thiobarbituric acid reactive substances (expressed in μM); (**B**) glutathione peroxidase (expressed as unit defined as the amount of enzyme that will cause the oxidation of 1.0 nmol of NADPH to NADP+ per minute at 25 °C); (**C**) superoxide dismutase (expressed as U/mL where one unit is the amount of enzyme needed to exhibit 50% dismutation of the superoxide radical) activities in plasma of lean Zucker rats (LZR black bar) and obese Zucker rats (OZR gray bar) at the age of 12, 16, and 20 weeks (X axis); (**D**) samples of plasma from LZR and OZR rats at different ages were immunoblotted using OxyBlot kit. Bar graph reports the values of optical density measured in optical density unit (ODU). Data are mean ± S.E.M. * *p* < 0.05 vs. age-matched LZR.

Analysis of oxidative condition was performed in plasma samples and in heart tissue in all groups of rats. The malondialdehyde (MDA) is the prototype of the so called thiobarbituric acid reactive substances (TBARS) [32]. MDA is also a product of lipid peroxidation and a marker of cell injury. It was significantly higher in the plasma of obese rats than in the control ones at all weeks of age (Figure 1A). However, there was no significant difference in the glutathione peroxidase (GPx) activity between lean and obese rats (Figure 1B), while the antioxidant activity of Superoxide dismutase (SOD) decreased in the plasma samples of OZRs compared to the LZRs (Figure 1C). The OxyBlot of plasma proteins showed an increase of oxidative state proteins in OZRs samples, particularly in the 20 weeks old rats (Figure 1D).

**Figure 2:**
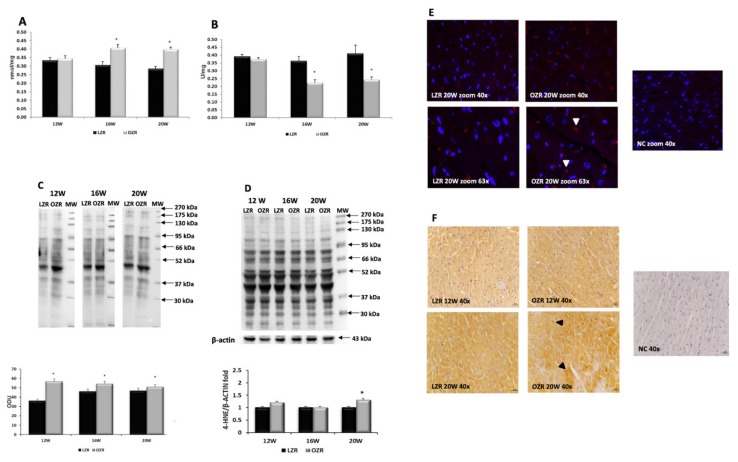
Oxidative stress in heart. (**A**) Concentration of thiobarbituric acid reactive substances (expressed in nmol/mg of tissue); (**B**) superoxide dismutase specific activity (expressed as U/mg of proteins where one unit is the amount of enzyme needed to exhibit 50% dismutation of the superoxide radical) in heart tissue of lean Zucker rats (LZR black bar) and obese Zucker rats (OZR grey bar) at the age of 12, 16, and 20 weeks (X axis); (**C**) lysates of heart from LZR and OZR rats were immunoblotted using the OxyBlot kit. Bar graph reports the values of optical density measured in optical density unit (ODU). Data are mean ± S.E.M. * *p* < 0.05 vs. age-matched LZR; (**D**) lysates of heart from LZR and OZR rats were immunoblotted using specific anti 4-Hydroxynonenal (4-HNE). Values indicate the ratio of densitometric analysis of bands and β-actin levels used as loading control, considering LZR group as reference. Blots are representative of one of three separate experiments; (**E**) sections of the heart of 20 weeks old LZR and OZR were processed for the immunohistochemistry of 8-oxo-dG at the magnification 40× zoom and 63× zoom. The immunoreaction is present more in the cytoplasm than nuclei of cells (arrow heads). NC: negative control; (**F**) sections of the heart of 12 and 20 weeks old LZR and OZR were processed for the immunohistochemistry of 4-HNE at the magnification 40×. The cardiomyocytes that are more immunoreactive are indicated with the arrow heads. NC: negative control. Calibration bar 25 µm.

According to the results in plasma, the MDA concentration in the heart tissues was significantly higher in OZRs compared to the LZRs, starting from 16 weeks of age (Figure 2A). In contrast, the SOD activity in the OZRs was diminished compared with LZRs (Figure 2B). The results of OxyBlot analysis showed an increase of oxidized proteins concentration in heart of obese animals (Figure 2C), while the expression of the lipid-aldehyde 4-hydroxynonenal (4-HNE), was slightly increased in these rats at 12 weeks of age and significantly increased in 20 weeks old OZRs (Figure 2D,F). The 8-oxo-2′-deoxyguanosine (8-oxo-dG) is used as a biomarker of oxidative DNA damage. Its immunofluorescence is weakly increased with aging, without a significant difference in intensity between the opposite experimental groups (Figure 2E). Figure 2E shows representative images of 8-oxo-dG staining more cytoplasm than nuclei.

The data of increased pro-oxidative elements and the decrease in the antioxidant properties revealed in OZRs a condition of oxidative stress—related to obesity.

### 2.3. Heart Morphology

**Figure 3:**
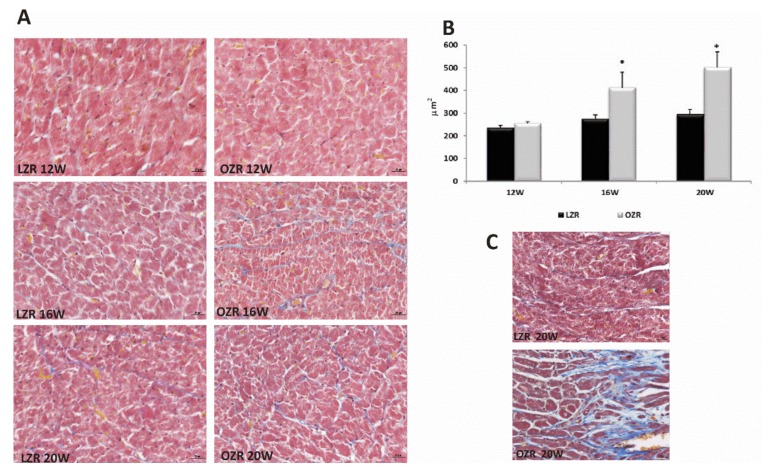
Heart morphology. (**A**) Ventricular sub-endocardium cardiomyocytes in heart tissue of lean Zucker rats (LZR) and obese Zucker rats (OZR) at the age of 12, 16, and 20 weeks, staining with Masson’s Trichrome technique. Magnification 40×. Calibration bar 25 µm; (**B**) morphometric analysis to evaluate the size of cardiomyocytes in 12, 16, and 20 weeks old LZR (black bar) and OZR (grey bar). Data are mean ± S.E.M. * *p* < 0.05 vs. age matched LZR; (**C**) apex of the heart stained with Masson’s trichrome of 20 weeks old lean Zucker rats (LZR) and obese Zucker rats (OZR). Magnification 40×. Calibration bar 25 µm.

The morphological (Figure 3A) and morphometric (Figure 3B) results showed a significant increase in the size of ventricular cardiomyocytes in the 16 and 20 weeks old OZRs compared to age-matched LZRs. Cardiac fibrosis characterized by an accumulation of extracellular matrix proteins and collagen deposition were found in subendocardial region at the level of the apex in the 20 weeks old obese rats (Figure 3C), but not in the younger rats [33].

### 2.4. Inflammation

**Figure 4:**
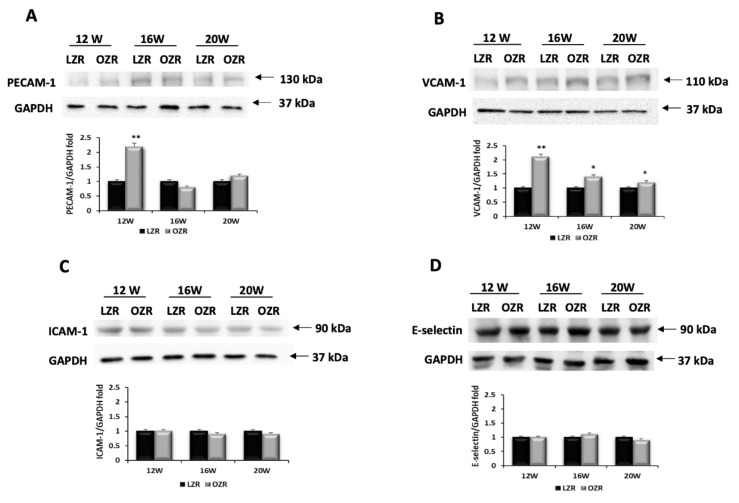
Inflammation. Lysates of heart from lean Zucker rats (LZR) and obese Zucker rats (OZR) at 12, 16, and 20 weeks of age, were immunoblotted using specific antibodies against: (**A**) platelet endothelial cell adhesion molecule-1 (PECAM-1); (**B**) vascular cell adhesion molecule-1 (VCAM-1); (**C**) intracellular adhesion molecule-1 (ICAM-1); (**D**) E-selectin. Values indicate the ratio of densitometric analysis of bands and GAPDH levels used as loading control, considering the LZR group as reference. Data are mean ± S.E.M. * *p* < 0.05 and ** *p* < 0.01 vs. age matched LZR. Blots are representative of one of three separate experiments.

**Figure 5:**
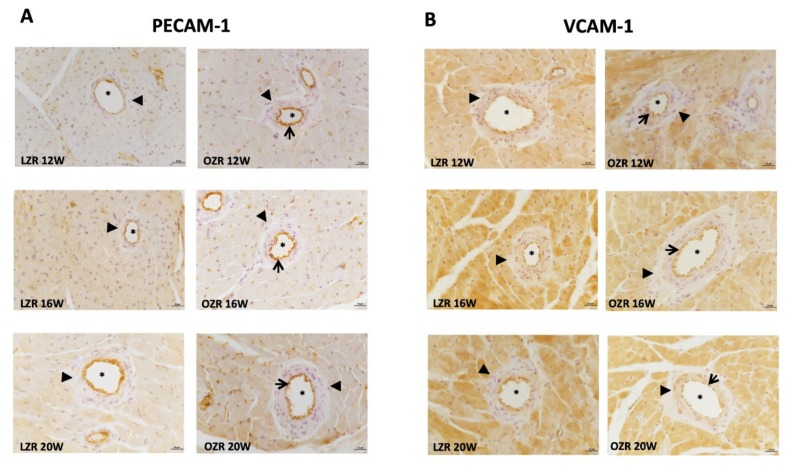
Inflammation. Sections of the heart of 12, 16, and 20 weeks old LZR and OZR processed for the immunohistochemistry of (**A**) platelet endothelial cell adhesion molecule-1 (PECAM-1); (**B**) vascular cell adhesion molecule-1 (VCAM-1), at the magnification 40×. The immunoreaction is present in the endothelium (arrows). The lumen of vessels is indicated with the asterisks, while the tunica media is negative (arrow heads). Calibration bar 25 µm.

Western blot, performed on heart tissue lysates, did not show a relevant change in PECAM-1 expression at 130 kDa and immunoreaction (Figure 4A and Figure 5A, respectively) except for 12 weeks old obese animals. The data reported a greater expression of VCAM-1 in OZRs at all ages (Figure 4B and Figure 5B), with a specific 110 kDa band (Figure 4B). Among the adhesion molecules studied, ICAM-1 was lowest expressed (Figure 4C), also confirmed by the immunohistochemistry (data not shown). Blood vessels were found positive for the others adhesion molecules (Figure 5A,B). It was also found that VCAM-1 was expressed in the cardiomyocytes (Figure 5B). In addition, the E-selectin was not modulated remarkably at 90 kDa (Figure 4D).

**Figure 6:**
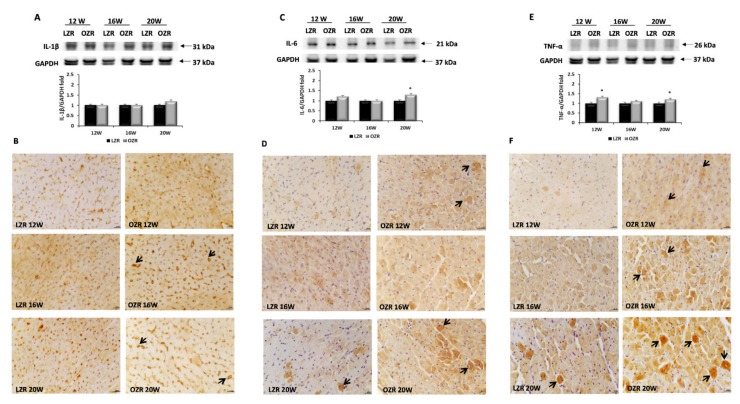
Inflammation. Lysates of heart from lean Zucker rats (LZR) and obese Zucker rats (OZR) at the age of 12, 16, and 20 weeks were immunoblotted using specific anti: (**A**) interleukin 1 beta (IL-1β); (**C**) interleukin 6 (IL-6); (**E**) tumor necrosis factor α (TNF-α). Values indicate the densitometric analysis using LZR rats as control. GAPDH levels were used as loading control. Data are mean ± S.E.M. * *p* < 0.05 vs. age matched LZR. Blots are representative of one of three separate experiments. TNF-α membrane was stripped and incubated with anti-IL-1β antibody. Control images were reused for illustrative purposes. Sections of the heart of 12, 16, and 20 weeks old LZR and OZR processed for the immunohistochemistry of (**B**) IL-1β; (**D**) IL-6; (**F**) TNF-α, at the magnification 40×. The cardiomyocytes that are more immunoreactive are indicated with the arrows. Calibration bar 25 µm.

Moreover, the expression of the cytokine IL-1β at 31 kDa was slightly increased only in the OZRs rats of 20 weeks of age in comparison to the age matched LZRs (Figure 6A,B). IL-6 was revealed with a specific 21 kDa band (Figure 6C); its expression was higher in 20 weeks old OZRs than in LZRs (Figure 6C,D). A similar trend was found for TNF-α with a band at 26 kDa (Figure 6E,F). The immunohistochemical analysis, performed for IL-1β, IL-6, and TNF-α, showed immunoreaction at the level of cardiomyocytes (Figure 6 B,D,F). In particular, the IL-1β immunoreaction was well defined in the external part of cardiomyocytes (Figure 6B), while the IL-6 and TNF-α expressions were present especially in the damaged cardiomyocytes (Figure 6D,F). The TNF-α immunoreaction was increased also with aging (Figure 6F).

The higher immunoreaction, shown for IL-6 and TNF-α in the older obese rats compared to lean, suggested an inflammatory process at the cardiovascular level due to obesity.

### 2.5. Cardiomyocytes Death Evaluation

**Figure 7:**
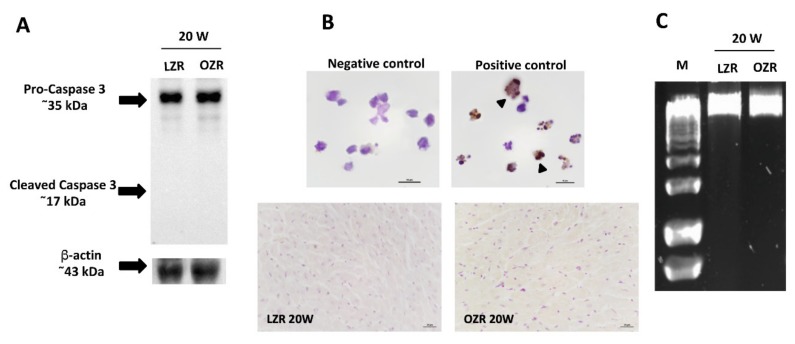
Apoptosis and necrosis. Lysates of the heart, obtained from LZR and OZR, at the age of 20 weeks, were immunoblotted using specific anti-caspase 3 proteases (**A**). β-actin levels were used as the loading control. Blots are representative of three separate experiments. Sections of the heart of 20 weeks old LZR and OZR were processed for the TUNEL. The specificity of the reaction was evaluated by using the negative and positive controls (head of the arrow) provided by kit (**B**). Calibration bar 25 µm. DNA ladder was absent in the samples of 20 weeks old LZR and OZR (**C**).

Based on our evidence of higher cardiomyocyte alterations in older OZRs, apoptosis was evaluated in the 20 weeks old LZRs and OZRs rats. No apoptotic or necrotic process was detected in the heart tissues of LZRs and OZRs as shown in Figure 7. In the Western blot analysis for caspase-3, only the band at about 35 kDa of uncleaved protease in LZRs and OZRs heart tissue was detected (Figure 7A). Furthermore, no TUNEL positive nuclei were observed in the sections of LZRs and OZRs heart samples (Figure 7B). Besides, no DNA degradation was present in the DNA samples extracted from the heart of LZRs and OZRs (Figure 7C).

## 3. Discussion

Although human obesity is not perfectly mimed by OZRs, these animals have been useful in understanding the causes and mechanisms that arise due to obesity and that contribute to the associated morbidity and mortality [34]. Moreover, OZR is commonly used to study the obesity related to type 2 diabetes. Indeed, the present study demonstrates that OZRs share many features with human MetS [35]: obesity, IR, hyperlipidemia, and elevated blood pressure [31].

It was previously demonstrated that, at 17 d, the OZRs eat more compared with LZRs and hyperphagia rises during the growth period (approximately 16 weeks of life). It was shown that the body composition of 14 weeks old obese Zucker rats, is 40% weight lipid. Moreover, the systolic blood pressure in 8–12 weeks old OZRs is lower than that in LZRs [30]. Based on this information, in our study, we sacrificed the animals at 12 weeks of age, when the OZRs demonstrated a condition of hyperphagia and bodyweight gain, but not yet the presence of overt hypertension. The difference in terms of blood pressure is not significant among the younger animals, but it was growing in obese phenotype, until reaching significance in 16 weeks old OZRs.

In humans, MetS are characterized by systemic oxidative stress [36,37] and high levels of proinflammatory cytokines [38,39]. Indeed, the adipose tissue secretes adipokines (someone with inflammatory function, such as IL-6) and these, in turn, generate ROS [40].

Previously, it was reported that OZRs exhibited an increase in oxidative stress [41,42]. As in our study, the MDA levels in the plasma and in the heart were significantly higher in older obese samples than in lean ones. The MDA content provided lipid peroxidation and peroxidative tissue injury, and more intense activity of pro-oxidant agents in aging OZRs, at the expense of antioxidant ones [43].

Besides, the SOD activity, as a major cellular defense system against superoxide, was decreased both in the plasma and in the heart of OZRs. Based on these results, the OZRs have a major risk of developing heart failure, because this disease has been reported to be related with oxidative stress in animal studies, with a concomitant lowering in antioxidant enzyme activity [44,45,46,47], and with antioxidant depletion in plasma [47] and heart [44,45,46] in animal studies.

Although the risk of cardiovascular events was inversely associated with increasing GPx activity [48], our results showed that GPx did not change in plasma samples. Thus, we can assess that GPx metabolic function was not affected as much as SOD metabolic activity.

It is important to highlight that the oxidative stress appeared in plasma of OZRs starting from 12 weeks of age. In the heart, oxidative stress was observed in 16 weeks old OZRs when a significant increase in size of cardiomyocytes was detected. This evidence confirms that cardiac hypertrophy was induced, not only by hypertension [49], but also by oxidative stress, as previously reported [50]. Morphological analysis showed cardiac fibrosis in older obese rats. Left ventricular hypertrophy, fibrosis, and impaired coronary flow reserve often accompanied cardiac diastolic dysfunction in obesity and aging [51].

Moreover, the OxyBlot and 4-HNE data showed an obvious increase of oxidative stress in the plasma and in the heart of obese rats. The increase in 4-HNE due to oxidative stress has been observed in several cardiac diseases, for example, diabetic cardiomyopathy. 4-HNE damages the myocardium by interfering with mitochondria and forming adducts [52].

Besides, high ROS production and decrease in antioxidant capacity lead to endothelial dysfunction, characterized by a reduction in the bioavailability of vasodilator nitric oxide (NO), and an increase in endothelium-derived contractile factors, leading to atherosclerosis [40]. Additionally, the increased stimulation of adhesion molecules, such as VCAM-1 in OZRs demonstrated the presence of endothelial activation and dysfunction strictly correlated with inflammation [53], as reported in subjects affected by the MetS [54], and obesity [16]. In our study, anti-VCAM-1 antibody also marked cardiomyocytes [55]. Our results showed that PECAM-1 expression was differently modulated. Indeed, the younger OZRs showed a remarkable increase of PECAM-1, however, the inflammation was reduced and then stabilized at 20 weeks of age, supposing a phenomenon of adaptation over time. The presence of hypertension in the OZRs also accompanied the endothelial dysfunction and the different modulation of the adhesion molecules [56]. There was an age-related increase in oxidative stress, but the inflammatory process seemed independent of age. This phenomenon may be explained by a direct correlation between inflammation and type II diabetes and (or) hyperlipidemia onset in younger OZRs.

Moreover, if circulating markers of inflammation, such as C-reactive protein, TNF-α, and some interleukins (for example, IL-6), are associated with propensity to develop ischemic [57,58] or atherosclerotic events [59], we hypothesize that OZRs might develop both of these complications. Besides, it was demonstrated that the high levels of TNF-α induce the production of ROS and lead to endothelial dysfunction in the MetS [60]. It is well known that IL-1β plays a role in many diseases related to MetS: type 2 diabetes, chronic heart failure, and atherosclerosis [61], however, we found slight differences in its expression between the older obese and lean Zucker rats. Although other researchers reported an increase of ICAM-1 and E-selectin in obesity [62], and in particular in the descending aorta of the 15 weeks old OZRs [63], our results showed no differences. This suggests that the expression of vascular adhesion protein (E-selectin), in obesity, may be modulated in the peripheral arteries rather than the coronary arteries.

However, it seems that fibrosis and hypertrophy of cardiomyocytes due to oxidative stress and inflammation, revealed for the first time by this study, were not enough to lead to a process of apoptosis or necrosis due to MetS in OZRs at least at 20 weeks of age.

Finally, our observations that were consistent with previous studies [64,65], confirmed that the increase of oxidative stress was associated with metabolic complications in OZRs, thus strongly correlated with MetS occurrence [66,67] and with the CVD pathogenesis [68,69]. Moreover, authors have demonstrated that an interrelation between inflammation and metabolic anomalies in type 2 diabetes can be a causal factor for vascular injury and it has been proposed that one indicator of these effects might be endothelial dysfunction in combination with a pro-coagulant state [70]. In the CVD, the main risk factor observed in patients, besides elevation of blood pressure and higher plasma lipids, is atherosclerosis, and it has since been convincingly verified that it is actually associated with inflammation [59,71].

Furthermore, these data represent the first report that clarifies the role of a concomitant presence of oxidative stress and the inflammatory markers in the heart of OZRs as an important link between MetS and CVD. However, other studies are necessary to identify the starting age when these phenomena are changing in obesity conditions and their possible correlation with weight gain, pericardial adipose tissue deposition, and blood parameters.

## 4. Materials and Methods

### 4.1. Experimental Animals

Male OZRs (*n* = 18) and their littermate lean Zucker rats (LZRs) (*n* = 18) were purchased from Harlan (Italy). The OZRs were studied starting from 12 weeks, when the MetS condition is not well-established yet, to 20 weeks of age. They were divided in three groups taking into consideration the age of sacrifice, performed at 12 weeks (*n* = 6, for each group), 16 weeks (*n* = 6, for each group), and 20 weeks of age (*n* = 6, for each group). The age of the sacrifice and the number of animals for each experimental group was calculated based on previous studies [27,28,72].

All animal experimental procedures were carried out in accordance with the Institutional Guidelines and complied with the Italian Ministry of Health (D. Lgs. 116/92–Art. 7) (Prot. N. 6198/2011) and associated guidelines from European Communities Council Directive (n. 86/609/CEE) governing animal welfare and protection.

The rats were housed starting from the 10th week of age and placed in single cages with a dark-light cycle of 12 h (light 07:00-19:00; dark 19:00-07:00). They were under standard diet (Mucedola 4RF18 mice and rats long term maintenance, containing 16% protein, 2.5% fat, and 7.5% max fiber and other nutritional additives) with the cage-free movement continues and food and water *ad libitum*. Food intake and body weight were monitored daily, while measurements of systolic blood pressure were performed once a week, in conscious rats, by tail-cuff methods using electronic sphygmomanometer, specific for small animals (Model: GIMA Italy, B3Plus). Before the sacrifice, in fasted rats, after systolic blood pressure measurement, blood withdrawals were performed from the tail vein. A total of 800 μL of blood was collected in tubes with l-heparin. The blood samples were then centrifuged for 10 min at 3000 rpm to measure the blood glucose, insulin, triglycerides, and total cholesterol. They were stored at 4 °C and delivered within 24 h to the “Fioroni” laboratory (San Benedetto del Tronto, AP, Italy) for the analysis. The heart was removed, and pericardial fatty tissue was completely removed. The heart was frozen at −80 °C for Western blot analysis or fixed in 4% paraformaldehyde and embedded in paraffin for morphological evaluations.

### 4.2. Morphological Aspects

Longitudinal heart sections (8 μm thick) were cut using a microtome, collected on slides and processed for morphological staining with Masson’s Trichrome. The sections were observed, and images were captured with the microscope by DS-Ri2 NIKON camera and evaluated using a NIS Elements Nikon image analyzer software (Nikon, Florence, Italy).

### 4.3. Western Blot

Protein lysate was obtained homogenizing the tissue (0.1 ± 0.02 g) in a Mixer Mill MM300 (Qiagen, Hilden, Germany) for 10 min, using lysis buffer containing Tris 1M pH 7.4, NaCl 1M, EGTA 10 mM, NaF 100 mM, Na_3_VO_4_ 100 mM, PMSF 100 mM, Deoxycholate 2%, EDTA 100 mM, Triton X100 10%, Glycerol, SDS 10%, Na_4_P_2_O_7_ 0.1M, Inhibitor Cocktail, distilled H_2_O. Equal amounts of protein (40 μg) were separated on 7%, 8%, 10%, and 12% SDS polyacrylamide gels, transferred onto nitrocellulose membranes and blotted with the specific antibodies. Non-specific binding sites were blocked with 5% BSA in PBS 0.1% Tween-20 for 1 h at room temperature. Optimal antibodies concentration was established through preliminary experiments. Membranes were incubated at 4°C overnight with the following primary antibodies: anti-ICAM-1 (Santa Cruz Biotechnology, Inc., Santa Cruz, CA, USA, 1:500), anti-VCAM-1 (Santa Cruz Biotechnology, Inc., Santa Cruz, CA, USA,1:500), anti-PECAM-1 (Santa Cruz Biotechnology, Inc., Santa Cruz, CA, USA, 1:500), anti-E-selectin (Santa Cruz Biotechnology, Inc., Santa Cruz, CA, USA, 1:500), anti-IL-1β (Santa Cruz Biotechnology, Inc., Santa Cruz, CA, USA, 1:200), anti-IL-6 (Santa Cruz Biotechnology, Inc., Santa Cruz, CA, USA, 1:200), anti-TNF-α (Santa Cruz Biotechnology, Inc., Santa Cruz, CA, USA, 1:500), anti-caspase-3 (Cell Signaling Technology, Danvers, MA, USA, 1:1000), anti-GAPDH (Cell Signaling Technology, Danvers, MA, USA, 1:1000) and anti-β-actin (Sigma-Aldrich Co., Merck KGaA, Darmstadt, Germany 1:3000) followed by incubation for 1 h at room temperature with corresponding HRP-conjugated donkey anti-goat, goat anti-rabbit, or goat anti-mouse secondary antibodies (BETHYL Laboratories, Inc., Montgomery, TX, USA, dilution 1:5000). The detection was performed using the LiteAblot PLUS kit (EuroClone, Milan, Italy). Band intensities were measured by densitometry with Nikon Imaging Software (NIS Elements) (Nikon, Florence, Italy) using GAPDH or β-actin as loading control. Blots are representative of one of three separate experiments.

### 4.4. Immunohistochemistry

Longitudinal sections of the heart 8 μm thick were cut using a microtome and collected on poly-l-lysine coated slides. After deparaffinization with xylene, the sections were hydrated through graded ethanol. Antigen retrieval was performed with a solution of Tris-EDTA pH 9. Endogenous peroxidase activity in the tissue was eliminated by a 20 min incubation with 3% H_2_O_2_, and nonspecific binding sites were blocked with BSA 3% in PBS-T for 1 h. The sections were exposed to the following primary antibodies diluted in PBS-T 0.3%: ICAM-1, VCAM-1, PECAM-1, IL1-β, IL-6 (all diluted 1:50), and TNF-α (diluted 1:100), overnight at 4 °C. The product of immune reaction was then revealed by exposing slides for 30 min at 25 °C with the specific biotinylated secondary antibodies: donkey anti-goat, goat anti-rabbit, or goat anti-mouse (BETHYL Laboratories, Inc., Montgomery, TX, USA) diluted 1:200 in PBS-T. The colored reaction product was developed with 3,3′-diamonobenzidine tetrahydrochloride (DAB) solution (Vector Laboratories, Inc., Burlingame, CA, USA). Some sections were incubated with a non-immune serum instead of a primary antibody to assess the background of immunostaining. Before dehydration in ethanol, sections were counterstained with hematoxylin.

### 4.5. TUNELAssay

Apoptotic nuclei were investigated in paraffin sections of the heart using a Calbiochem^®^ DNA Fragmentation kit (Millipore Merck, Darmstadt, Germany). following the related protocol provided by the company. Shortly, terminal deoxynucleotidyl transferase (TdT) binds to exposed 3′-OH ends of DNA fragments and catalyzes the addition of biotin-labeled unlabeled deoxynucleotides. Biotinylated nucleotides are detected using streptavidin-horseradish peroxidase conjugate. DAB reacts with the labeled sample to generate an insoluble colored substrate in the fragmented nuclei. The sections were counterstained for 20 s with a hematoxylin solution.

### 4.6. DNA Ladder Evaluation

DNA degradation was investigated in heart tissue, after extraction of DNA, using a specific Apoptotic DNA Ladder Kit (Roche Diagnostics GmbH, Mannheim, Germany), and following the specific procedures provided by the kit. The extracted DNA was loaded in a 1.7% agarose gel.

### 4.7. Biomarkers of Oxidative Stress

Different oxidative stress indicators were evaluated in plasma samples and in heart homogenates. Lipid peroxidation was quantified by measuring the accumulation of TBARS (Cayman, Chemical Company, Ann Arbor, MI, USA, Cat. No. 10009055) in homogenates and expressed as MDA content. The content of MDA was measured at 532 nm using the method described by the company; SOD activity by Cayman, Chemical Company (Ann Arbor, MI, USA), Cat. No. 706002 and GPx activity by Cayman, Chemical Company (Ann Arbor, MI, USA), Cat. No. 703102 were also evaluated. The protein oxidation status was investigated using the OxyBlot Protein Oxidation Detection Kit (Merk, Millipore, Burlington, MA, USA, Cat. No. S7150). The expression of a biomarker for oxidative stress, 4-HNE (Santa Cruz Biotechnology, Inc., Santa Cruz, CA, USA), was evaluated both through Western blot (dilution 1:500), normalizing the band intensity with the loading control β-Actin (Sigma-Aldrich Merck, KGaA, Darmstadt, Germany, MO, USA, 1:3000), and through immunohistochemistry (dilution 1:250). Moreover, a mouse monoclonal antibody specific for 8-oxo-dG (TREVIGEN, Gaithersburg, MD, USA, Cat. No. 4354-MC-050), the major oxidative DNA damage product, was used according to the manufacturer’s protocol. After the incubation with the goat anti-mouse secondary antibody (Alexa Fluor 488), the sections were counterstained with 4′,6-Diamidino-2-Phenylindole, Dihydrochloride (DAPI) and were viewed using a Nikon mod.C2 plus Confocal Laser Microscope (Nikon Imaging Japan Inc., Japan).

### 4.8. Statistical Analysis

Means of different parameters investigated were calculated from single animal data, and group means ± S.E.M., were then derived from single animal values. The significance of differences between means was estimated by analysis of variance (ANOVA) followed by the Bonferroni multiple range tests, setting *p* < 0.05 value as a significant difference.

## Figures and Tables

**Table 1:** Physiological and blood parameters of obese Zucker rats (OZR) and lean Zucker rats (LZR) at 12, 16, and 20 weeks of age.

Parameter	12 Weeks		16 Weeks		20 Weeks	
	LZR	OZR	LZR	OZR	LZR	OZR
Body weight (g)	284.7 ± 6.1	411.3 ± 5.4 *	356.1 ± 6.2	519.8 ± 7.8 *	377.1 ± 8.7	566.2 ± 19.6 *
24 h Food intake (g)	26.8 ± 0.9	36.7 ± 1.5 *	24.4 ± 0.7	31.1 ± 0.7 *	25.6 ± 1.8	33.2 ± 3.1 *
Systolic blood pressure (mmHg)	103.6 ± 9.1	120.1 ± 13.7	104.3 ± 6.4	140.8 ± 5.6 *	99.8 ± 2.2	137.3 ± 4.2 *
Weight of the heart (g)	1.02 ± 0.04	1.18 ± 0.02 *	1.14 ± 0.05	1.32 ± 0.02 *	1.15 ± 0.03	1.31 ± 0.04 *
Glucose (mmol/l)	5.28 ± 0.26	6.56 ± 0.21 *	4.47 ± 0.25	6.22 ± 0.40 *	5.22 ± 0.15	6.39 ± 0.30 *
Insulin (pmol/l)	5.2 ± 1.7	444.8 ± 65.6 *	13.8 ± 3.4	751.7 ± 63.8 *	12.1 ± 1.7	598.3 ± 44.8 *
Triglycerides (mmol/l)	0.53 ± 0.02	3.69 ± 0.33 *	0.54 ± 0.03	3.91 ± 0.33 *	0.65 ± 0.07	4.55 ± 0.36 *
Total cholesterol (mmol/l)	2.33 ± 0.05	3.76 ± 0.13 *	2.2 ± 0.10	2.58 ± 0.26 *	2.58 ± 0.08	5.09 ± 0.22 *
LDL cholesterol (nmol/dl)	0.28 ± 0.02	0.22 ± 0.01	0.19 ± 0.01	0.24 ± 0.02 *	0.27 ± 0.02	0.40 ± 0.03 *
HDL cholesterol (nmol/dl)	0.82 ± 0.03	1.16 ± 0.04 *	0.65 ± 0.02	0.98 ± 0.02 *	0.78 ± 0.02	1.09 ± 0.02 *

Data are mean ± S.E.M. * *p*< 0.05 vs. age-matched LZR.

## Abbreviations

4-HNE	4-hydroxynonenal
8-oxo-dG	8-oxo-2′-deoxyguanosine
CVD	cardiovascular diseases
GPx	glutathione peroxidase
ICAM-1	intercellular cell adhesion molecule-1
IL-1β	interleukin-1β
IL-6	interleukin-6
IR	insulin resistance
LZRs	lean Zucker rats
MDA	malondialdehyde
MetS	metabolic syndrome
OZRs	obese Zucker rats
PECAM-1	platelet cell adhesion molecule-1
SOD	superoxide dismutase
TBARSTdT	thiobarbituric acid reactive substancesterminal deoxynucleotidyl transferase
TNF-α	tumor necrosis factor-α
VCAM-1	vascular cell adhesion molecule-1
